# Dietary background, serum polyunsaturated fatty acid profiles, and 1-year outcomes after large-artery atherosclerotic stroke: a multicenter cohort study

**DOI:** 10.3389/fneur.2026.1864966

**Published:** 2026-07-10

**Authors:** Xiaoci Lin, Zhihao Zhao, Daijun Zhu, Guogu Shen, Xuri Xu, Yinyan Li

**Affiliations:** 1 Department of Neurology, First People's Hospital of Daishan County, Zhoushan, Zhejiang, China; 2 Department of Nutrition, First People's Hospital of Daishan County, Zhoushan, Zhejiang, China

**Keywords:** dietary pattern, large-artery atherosclerotic cerebral infarction, neuropsychiatric outcomes, polyunsaturated fatty acids, post-stroke prognosis, stroke outcomes, ω-3 fatty acids, ω-6 fatty acids

## Abstract

**Objective:**

To examine whether long-term dietary background is associated with post-stroke outcomes in patients with large-artery atherosclerotic cerebral infarction (LAA-CI), and to explore the relationship between serum polyunsaturated fatty acid (PUFA) profiles and neurological, functional, and neuropsychiatric recovery.

**Methods:**

This retrospective multicenter cohort study enrolled 410 patients with LAA-CI admitted to the neurology departments of two hospitals in Zhejiang Province, China, between June 2022 and December 2024. Patients were stratified by long-term dietary background into a high-fish/seafood dietary group (*n* = 210) and a mixed/omnivorous inland dietary group (*n* = 200). All patients underwent 1-year follow-up with assessment of modified Rankin Scale (mRS) score, poor functional outcome (mRS > 2), change in NIHSS score (ΔNIHSS), post-stroke depression (HAMD), and post-stroke cognitive impairment (MMSE). In a representative subgroup (*n* = 100), serum PUFA profiling was performed by gas chromatography-mass spectrometry. Multivariable logistic and linear regression models and ordinal trend analyses were applied to quantify associations between dietary background, PUFA indices, and clinical outcomes.

**Results:**

Baseline demographic and major clinical characteristics were comparable between the two groups. At 1-year follow-up, the island group showed lower mRS scores, a lower incidence of poor functional outcomes, and greater neurological improvement (all *P* < 0.05), along with lower rates of post-stroke depression and cognitive impairment (all *P* < 0.01) compared with the inland group. In the PUFA subgroup, the island group exhibited higher ω-3 levels and lower ω-6/ω-3 ratios (both *P* < 0.001). Further analyses demonstrated that higher ω-6/ω-3 ratios were associated with increasing risks of poor functional outcome, post-stroke depression, and cognitive impairment (all *P* for trend < 0.05), and were negatively correlated with ΔNIHSS (β = −0.34, *P* = 0.003).

**Conclusion:**

Differences in long-term dietary background are associated with heterogeneity in post-stroke recovery. The ω-6/ω-3 ratio may represent a metabolic marker linked to functional, neurological, and neuropsychiatric outcomes after stroke, with potential implications for risk stratification and follow-up management, providing a novel research direction for post-stroke risk stratification and follow-up management.

## 1 Introduction

Stroke remains a leading cause of long-term disability worldwide, with large-artery atherosclerotic cerebral infarction (LAA-CI) representing a major etiological subtype in Asian populations (1, 2). Despite advances in acute management and secondary prevention, substantial heterogeneity persists in post-stroke functional recovery, as well as in cognitive and emotional outcomes (3, 4). Identifying modifiable factors associated with post-stroke prognosis has therefore become a critical focus in stroke research. Dietary factors are increasingly recognized as important contributors to vascular risk and cardiometabolic health (5, 6). However, their role in post-stroke recovery remains less clearly defined. Previous studies have largely focused on general populations or primary prevention, often relying on self-reported dietary assessments or broad dietary pattern classifications, which limits the ability to link dietary exposure to biological mechanisms and clinical outcomes in specific stroke subtypes (7, 8).

Long-term dietary patterns may influence stroke outcomes through alterations in metabolic profiles, particularly polyunsaturated fatty acids (PUFAs) (9, 10). The balance between ω-6 and ω-3 fatty acids has been implicated in inflammatory regulation, vascular function, lipid metabolism, and neuronal recovery (11, 12). Nevertheless, evidence regarding their association with post-stroke outcomes in patients with large-artery atherosclerotic stroke remains limited, and the prognostic relevance of serum PUFA profiles in this specific population has not been fully clarified (13). In this context, the present study aimed to investigate the relationship between dietary background and post-stroke clinical outcomes, and to further evaluate whether serum PUFA profiles, especially the ω-6/ω-3 ratio, are associated with functional, neurological, and neuropsychiatric recovery.

## 2 Methods

### 2.1 General information

This study adopted a retrospective multicenter cohort design. A total of 410 patients with ischemic stroke were consecutively enrolled from the neurology departments of two tertiary hospitals in Zhejiang Province, China, between June 2022 and December 2024: Daishan County First People's Hospital contributed 210 patients and Zhejiang Provincial Hospital of Traditional Chinese Medicine contributed 200 patients. All patients were diagnosed with ischemic stroke on the basis of cranial magnetic resonance imaging or computed tomography and were classified as LAA-CI according to the TOAST criteria; the time of stroke onset and admission assessment was defined as the baseline (T0) (14). Patients were stratified by their long-term, pre-stroke dietary background into two groups. The high-fish/seafood dietary group (island group) comprised individuals with long-term residence in coastal or island areas whose habitual diet was predominantly based on fish and other marine products as the primary protein source. The omnivorous inland dietary group (inland group) comprised individuals with long-term residence in inland areas whose diet was predominantly based on livestock, poultry, and plant-based foods. Dietary background was ascertained retrospectively from electronic medical records and previously completed food-frequency questionnaires; only patients with stable, unchanged residence for at least 3 years prior to stroke onset and with complete dietary information were included. Dietary background was determined comprehensively on the basis of previous medical records, dietary survey data, and the predominant source of dietary protein, and group allocation was completed before statistical analysis. Serum samples collected at admission were stored for subsequent PUFA analysis. All patients had complete clinical data and 1-year follow-up records. Inclusion criteria were: (1) age 18 years or older; (2) imaging-confirmed ischemic stroke meeting TOAST criteria for LAA-CI; (3) admission NIHSS score of 25 or below; (4) anterior-circulation infarction involving the internal carotid artery, middle cerebral artery, or anterior cerebral artery territory, confirmed by clinical and imaging findings; (5) no prior use of fatty acid supplements or PUFA-related medications; and (6) stable residence in the designated recruitment area for at least 3 years before stroke onset. Exclusion criteria were: (1) severe hepatic or renal dysfunction, gastrointestinal malabsorption, or other systemic conditions affecting fatty acid metabolism; (2) failure to meet the geographic or dietary stability criteria for the assigned group; (3) unavailability of dietary history data; and (4) pre-existing diagnosis of depression or cognitive impairment, or a psychiatric disorder precluding follow-up assessment. No study-specific interventions were performed and all data were derived from routine clinical records. Both centers maintained standardized protocols for stroke diagnosis, treatment, and laboratory testing to minimize inter-site variability.

### 2.2 Data processing

All study variables were subjected to data completeness and plausibility checks prior to statistical analysis, including identification of outliers and verification of logical consistency. Continuous variables were assessed using descriptive statistics and distribution tests to evaluate their distribution characteristics, and apparent outliers were verified against the original medical records. For missing data, the proportion and distribution of missingness for each variable were first evaluated. If key baseline variables or primary outcome indicators were missing, the corresponding cases were not included in the relevant statistical analyses. For variables with a low proportion of missing data, complete-case analysis was applied, and only cases with complete information on the relevant variables were included in the analysis.

### 2.3 Data collection

Baseline data were obtained through retrospective review of the electronic medical record systems and follow-up records of the two hospitals. All baseline clinical characteristics and laboratory indicators were referenced to the time point of stroke onset and admission assessment (T0), and laboratory indicators were derived from the first blood sample collected after admission.

#### 2.3.1 Demographic data

Demographic data included age, sex, and body mass index (BMI), which were used to describe the basic characteristics of the study population and were incorporated as potential confounding factors into subsequent statistical analyses.

#### 2.3.2 Stroke-related clinical characteristics

Data on age at stroke onset, blood pressure at admission, degree of neurological deficit at admission, and imaging characteristics were collected. The degree of neurological deficit was assessed using the National Institutes of Health Stroke Scale (NIHSS); etiological classification of ischemic stroke was determined according to the TOAST classification criteria; imaging data were used to determine the location of cerebral infarction and the responsible vessel.

#### 2.3.3 Past medical history and concomitant medication

The medical history information included hypertension, diabetes mellitus, coronary heart disease, atrial fibrillation, and hyperlipidemia. Medication use data included antiplatelet agents, statins, antihypertensive drugs, and antidiabetic drugs administered before stroke onset and after hospital discharge. Information on acute-phase reperfusion therapy, smoking history, and alcohol consumption history was also collected. Reperfusion therapy included intravenous thrombolysis and endovascular thrombectomy. Smoking history was defined as a previous or current pattern of regular tobacco use. Alcohol consumption history was defined as a previous or current pattern of regular alcohol intake. Discharge destination was categorized as home, rehabilitation facility, or nursing/care institution. Educational attainment was categorized as primary school or below, and junior high school, senior high school, or above.

#### 2.3.4 Baseline laboratory indicators

Laboratory indicators included lipid-related parameters: total cholesterol (TC), triglycerides (TG), low-density lipoprotein cholesterol (LDL-C), and high-density lipoprotein cholesterol (HDL-C); glucose metabolism-related parameters: fasting plasma glucose (FPG) and glycated hemoglobin (HbA1c); as well as uric acid (UA).

#### 2.3.5 Diet-related factors

Dietary information was retrospectively compiled using a standardized dietary questionnaire uniformly adopted by both hospitals. The questionnaire was primarily designed to assess the stable long-term dietary habits established before stroke onset. Both hospitals used the same version of the questionnaire and followed a standardized survey procedure. Information was verified by trained healthcare personnel to improve the consistency and reliability of dietary data collection. The questionnaire included dietary information related to fatty acid intake patterns, including the frequency of fish and other seafood consumption, red meat consumption, soybean and soy product consumption, pickled food consumption, fried food consumption, and the primary type of cooking oil used. The frequency of food consumption was recorded using a graded scale (5–7 days/week, 3–4 days/week, 1–2 days/week, and < 1 day/week) and was quantitatively assessed in combination with the average daily intake. Long-term dietary background was comprehensively determined on the basis of previous medical records, dietary survey data, stable residence information over the preceding 3 years, and the predominant source of dietary protein. The island group was defined as individuals who had resided long-term in island areas and whose primary dietary protein source was fish and other seafood products. The inland group was defined as individuals who had resided long-term in inland areas and whose dietary pattern was predominantly based on livestock and poultry meat as well as plant-based foods.

### 2.4 Measurement of polyunsaturated fatty acid profiles

All included patients had fasting peripheral venous blood collected on the morning of the day following admission, and serum samples were stored. Among patients meeting the sample quality requirements and having complete follow-up data, a stratified random sampling method was used to select 50 patients from the island group and 50 patients from the inland group, yielding a total of 100 patients for serum polyunsaturated fatty acid (PUFA) profile analysis. The remaining patients (*n* = 310) were not included in the planned sampling framework. The sampling process was conducted independently of outcome assessment and was completed before statistical analysis. A total of 5 mL of fasting peripheral venous blood was collected in the morning of the day following admission. After centrifugation to separate serum, samples were stored at low temperature and transported to the same laboratory for unified analysis to minimize batch effects and inter-laboratory variability. All samples underwent quality assessment prior to analysis, and those with hemolysis or not meeting storage requirements were excluded. The serum PUFA profile was analyzed using gas chromatography–mass spectrometry, with an Agilent 7,890B−7,000D gas chromatograph coupled with a tandem mass spectrometry system. The analytical procedures were performed strictly in accordance with the instrument operating protocols and reagent instructions. All samples were analyzed either in the same batch or in batches following standardized procedures, and all analyses were conducted by trained technical personnel. Commercial assay kits (MERCK, Germany) were used for PUFA detection. The measured ω-3 PUFAs mainly included Eicosapentaenoic Acid (EPA), Docosapentaenoic Acid (DPA), and Docosahexaenoic Acid (DHA), while ω-6 PUFAs mainly included Linoleic Acid (LA), Arachidonic Acid (AA), and γ-linolenic acid (GLA). The levels of each fatty acid were expressed as mass concentrations. Based on these measurements, the ratio of serum ω-6 to ω-3 PUFAs was calculated as a composite indicator reflecting the overall fatty acid composition and balance *in vivo*. Referring to previous studies and relevant nutritional recommendations, ω-6/ω-3 ratio thresholds of 4 and 10 were used as stratification cutoffs to categorize the study population into low, medium, and high groups, which were subsequently used for outcome risk comparisons and trend analyses (15). As serum polyunsaturated fatty acid profile analysis was performed in only a subset of patients, the related analyses were defined as exploratory subgroup analyses and were primarily conducted to evaluate potential associations between fatty acid profile characteristics and clinical outcomes.

### 2.5 Outcome indicators

#### 2.5.1 Modified Rankin Scale (mRS)

Used to assess overall functional recovery after stroke. The mRS is a widely used clinical tool for evaluating functional outcomes, with a score range of 0–6, where 0 indicates no symptoms, 1–2 indicates slight disability but ability to live independently, 3–5 indicates varying degrees of functional dependence, for patients who died during follow-up, the mRS score was recorded as 6. The time of stroke onset and admission assessment was defined as the baseline time point (T0), and follow-up assessment was conducted at 1 year after stroke. An mRS score >2 at follow-up was defined as a poor functional outcome, which was used to compare long-term functional outcomes among patients with different dietary backgrounds and fatty acid profile characteristics (16).

#### 2.5.2 National Institutes of Health Stroke Scale (NIHSS)

Used to assess the severity of neurological deficits in patients with stroke. The scale consists of 15 items, with a total score ranging from 0 to 42, where higher scores indicate more severe neurological impairment. The NIHSS score at the time of stroke onset and admission assessment was used as the baseline level (T0), and follow-up assessment was conducted at 1 year after stroke. The change in NIHSS score from baseline to follow-up (ΔNIHSS) was calculated as the NIHSS score at follow-up minus the baseline NIHSS score (ΔNIHSS = NIHSS_follow-up—NIHSS_baseline), with lower values indicating greater improvement in neurological function. This measure was used to reflect the magnitude of neurological recovery or improvement after stroke (17).

#### 2.5.3 Mini-Mental State Examination (MMSE)

The MMSE, proposed by Folstein et al., was used to assess overall cognitive function. The total score is 30 points and covers six cognitive domains, including orientation, memory, attention and calculation, language, and visuospatial ability. Lower scores indicate more severe cognitive impairment. Patients who were unable to complete the assessment because of aphasia or severe functional impairment were excluded from the outcome analysis. The MMSE score obtained during the post-stroke follow-up period was used as the basis for evaluation. The occurrence of post-stroke cognitive impairment was determined according to the MMSE score completed during follow-up and was used to analyze the association between different fatty acid profile characteristics and post-stroke cognitive impairment.

#### 2.5.4 Hamilton Depression Rating Scale (HAMD)

Proposed by Hamilton, the HAMD was used to assess the severity of depressive symptoms. It is a commonly used clinical tool for evaluating depression, consisting of 17 items, with some items scored on a 0–4 scale and others on a 0–2 scale, yielding a total score range of 0–52. Higher scores indicate more severe depressive symptoms. Generally, a HAMD score ≤ 7 indicates no depression, 8–16 indicates mild depression, 17–23 indicates moderate depression, and ≥24 indicates severe depression. Patients who were unable to complete the assessment because of aphasia or severe functional impairment were excluded from the outcome analysis. The occurrence of post-stroke depression was determined based on HAMD scores recorded in previous medical records and follow-up data during the post-stroke follow-up period, and was used to compare emotional outcomes among patients with different fatty acid profile levels.

Stroke recurrence was used as an event outcome indicator to assess the long-term risk of cerebrovascular events. Stroke recurrence was defined as the occurrence of new acute neurological deficit symptoms during the follow-up period, confirmed by imaging as a new ischemic or hemorrhagic stroke event. Taking the index stroke as the starting point, the occurrence of stroke recurrence events was recorded during the 1-year follow-up period after stroke and was used for subsequent risk comparison analysis.

All follow-up outcomes were assessed by neurologists or trained research personnel who had received standardized training and followed uniform assessment procedures. Outcome assessors were not involved in the classification of dietary background and had no access to information regarding patients' dietary group assignments during the assessment process. The mRS, NIHSS, HAMD, and MMSE were all evaluated using standardized scoring criteria.

### 2.6 Statistical analysis

Statistical analyses were performed using SPSS 19 and R 4.2.1 software. Continuous variables were presented as mean ± standard deviation (x ± s) according to their distribution characteristics, and between-group comparisons were conducted using the *t*-test or Mann-Whitney *U*-test, as appropriate. Categorical variables were expressed as number of cases and percentages, and between-group comparisons were performed using the χ^2^-test or Fisher's exact test. The primary outcome analyses were conducted in the full study population. Binary outcomes were analyzed using logistic regression models, and the continuous outcome (ΔNIHSS) was analyzed using a linear regression model. Regression results were presented as ORs or regression coefficients (β) with their 95% confidence intervals, respectively. The mRS score was treated as an ordinal categorical variable and analyzed using an ordinal logistic regression sensitivity analysis based on the proportional odds model. The ω-6/ω-3 ratio was entered into the multivariable regression models as a continuous variable. Analyses related to serum polyunsaturated fatty acids were conducted in the fatty acid detection subgroup. Serum ω-3, ω-6, and the ω-6/ω-3 ratio were standardized in the regression analyses per 1 standard deviation increase. In addition, a linear regression model was further used to analyze the association between the serum polyunsaturated fatty acid profile and the magnitude of neurological improvement (ΔNIHSS). In the grouped analysis of the ω-6/ω-3 ratio, the groups were entered into the model as an ordinal variable, and trend tests were used to evaluate changes in outcome risk with increasing ratio levels. Poor functional outcome, post-stroke depression, and post-stroke cognitive impairment were prespecified as the three primary outcome measures. Each outcome was proposed independently on the basis of biological mechanisms related to stroke prognosis. Because the above subgroup analyses were predefined as exploratory analyses, no additional adjustment for multiple comparisons was performed, and all statistical results were reported in full. Multivariable models were uniformly adjusted for age, sex, body mass index, admission NIHSS score, hypertension, diabetes mellitus, coronary heart disease, atrial fibrillation, hyperlipidemia, acute-phase reperfusion therapy, secondary prevention medications after discharge, smoking history, alcohol consumption history, discharge destination, and educational attainment. In addition, to control for the potential impact of center differences, the source hospital was included in the model as a covariate in the sensitivity analysis. All statistical tests were two-sided, and *P* < 0.05 was considered statistically significant.

## 3 Results

### 3.1 Comparison of baseline characteristics and dietary background grouping of the study population

A total of 410 patients with large-artery atherosclerotic cerebral infarction were included, including 210 cases in the island group and 200 cases in the inland dietary background group. The mean age of the study population was 72.8 ± 8.2 years, with 246 males (60.0%). The median NIHSS score at admission was 7.0 (5.0–9.0), and approximately 70.0% of patients had the responsible vessel located in the internal carotid artery system. Among major cerebrovascular risk factors, the prevalence rates of hypertension, diabetes mellitus, hyperlipidemia, coronary heart disease, and atrial fibrillation were 75.1, 33.9, 46.1, 24.4, and 14.9%, respectively. There were no statistically significant differences between the two groups in age, sex distribution, body mass index, admission NIHSS score, type of responsible vessel, or major cerebrovascular risk factors (all *P* > 0.05), indicating good comparability of baseline characteristics between the two groups. In terms of laboratory indicators, compared with the inland group, patients in the island group had lower triglyceride levels and higher high-density lipoprotein cholesterol levels (both *P* < 0.05), while no statistically significant differences were observed in other indicators (all *P* > 0.05). See Table 1. To assess the representativeness of the fatty acid detection subgroup, further comparisons were conducted between this subgroup and the overall cohort in terms of major baseline characteristics and outcome distributions, and no statistically significant differences were found (all *P* > 0.05). See Supplementary Table 1. The distribution of dietary groups was highly correlated with the source hospital. The distribution of dietary group assignments across centers is presented in Supplementary Table 2.

**Table 1:** Comparison of baseline characteristics of patients with large-artery atherosclerotic cerebral infarction (*n* = 410).

Variable	Overall (*n* = 410)	Island group (*n* = 210)	Inland group (*n* = 200)	*t*/*z*/χ^2^	*P* value
Age (years)	72.8 ± 8.2	73.1 ± 8.0	72.5 ± 8.4	0.68	0.497
Sex (male), *n* (%)	246 (60.0%)	130 (61.9%)	116 (58.0%)	0.64	0.423
BMI (kg/m^2^)	24.3 ± 3.2	24.1 ± 3.0	24.5 ± 3.4	−1.18	0.239
Educational attainment				6.24	0.044
Primary school or below, *n* (%)	148 (36.1%)	86 (41.0%)	62 (31.0%)		
Junior high school, *n* (%)	158 (38.5%)	80 (38.1%)	78 (39.0%)		
Senior high school or above, *n* (%)	104 (25.4%)	44 (21.0%)	60 (30.0%)		
Admission NIHSS score	7.0 (5.0–9.0)	7.0 (5.0–9.0)	7.0 (5.0–10.0)	−0.72	0.472
Acute-phase reperfusion therapy, *n* (%)	82 (20.0%)	36 (17.1%)	46 (23.0%)	2.16	0.142
Responsible vessel: internal carotid artery system, *n* (%)	287 (70.0%)	142 (67.6%)	145 (72.5%)	1.14	0.285
History of hypertension, *n* (%)	308 (75.1%)	160 (76.2%)	148 (74.0%)	0.29	0.592
History of diabetes mellitus, *n* (%)	139 (33.9%)	72 (34.3%)	67 (33.5%)	0.03	0.864
History of coronary heart disease, *n* (%)	100 (24.4%)	50 (23.8%)	50 (25.0%)	0.07	0.793
History of atrial fibrillation, *n* (%)	61 (14.9%)	32 (15.2%)	29 (14.5%)	0.04	0.845
History of hyperlipidemia, *n* (%)	189 (46.1%)	96 (45.7%)	93 (46.5%)	0.03	0.872
Smoking history, *n* (%)	156 (38.0%)	82 (39.0%)	74 (37.0%)	0.17	0.678
Alcohol consumption history, *n* (%)	102 (24.9%)	56 (26.7%)	46 (23.0%)	0.71	0.399
Pre-stroke antiplatelet use, *n* (%)	123 (30.0%)	62 (29.5%)	61 (30.5%)	0.04	0.843
Pre-stroke statin use, *n* (%)	119 (29.0%)	58 (27.6%)	61 (30.5%)	0.43	0.511
Post-discharge antiplatelet therapy, *n* (%)	368 (89.8%)	188 (89.5%)	180 (90.0%)	0.02	0.876
Post-discharge statin therapy, *n* (%)	352 (85.9%)	179 (85.2%)	173 (86.5%)	0.12	0.727
Discharge destination				4.38	0.112
Home, *n* (%)	298 (72.7%)	158 (75.2%)	140 (70.0%)		
Rehabilitation facility, *n* (%)	88 (21.5%)	40 (19.0%)	48 (24.0%)		
Nursing/care institution, *n* (%)	24 (5.9%)	12 (5.7%)	12 (6.0%)		
TC (mmol/L)	4.82 ± 0.96	4.74 ± 0.93	4.90 ± 0.98	−1.64	0.102
TG (mmol/L)	1.48 (1.06–1.96)	1.35 (1.00–1.72)	1.62 (1.15–2.15)	−2.68	0.007
LDL-C (mmol/L)	2.83 ± 0.79	2.76 ± 0.77	2.91 ± 0.81	−1.86	0.064
HDL-C (mmol/L)	1.15 ± 0.27	1.20 ± 0.28	1.11 ± 0.26	2.87	0.004
FPG (mmol/L)	6.15 ± 1.71	6.03 ± 1.63	6.27 ± 1.79	−1.34	0.182
HbA1c (%)	6.41 ± 1.32	6.21 ± 1.22	6.61 ± 1.41	2.16	0.031
UA (μmol/L)	339.2 ± 90.1	332.1 ± 88.0	346.6 ± 92.3	−1.62	0.106

### 3.2 Serum polyunsaturated fatty acid profile characteristics in patients with different dietary backgrounds

In the fatty acid detection subgroup, the island group exhibited higher ω-3 levels, lower ω-6 levels, and a lower ω-6/ω-3 ratio (all *P* < 0.05). See Table 2.

**Table 2:** Comparison of serum polyunsaturated fatty acid profile characteristics among patients with different dietary backgrounds (fatty acid detection subgroup, *n* = 100).

Indicator	Island group (*n* = 50)	Inland group (*n* = 50)	*z* value	*P* value
ω-3 polyunsaturated fatty acids (μg/mL)	448.9 (372.6–535.4)	372.8 (308.9–456.1)	−3.84	< 0.001
ω-6 polyunsaturated fatty acids (μg/mL)	1782.6 (1559.8–2014.7)	1876.9 (1651.2–2118.5)	−2.16	0.031
ω-6/ω-3 ratio	3.98 (3.31–4.85)	4.93 (4.09–5.98)	−4.21	< 0.001

### 3.3 Follow-up functional, neurological, and event outcomes in patients with different dietary backgrounds

Compared with the inland group, patients in the island group had lower overall mRS scores at 1 year [2.0 (1.0–3.0) vs. 3.0 (2.0–4.0), *P* < 0.001], and a significantly lower incidence of poor functional outcomes (mRS>2) (30.5% vs. 43.5%, *P* = 0.008). Meanwhile, the island group showed an overall trend toward lower follow-up NIHSS scores, with a greater magnitude of neurological improvement, and the differences were statistically significant (all *P* < 0.05). In addition, the incidences of post-stroke depression and post-stroke cognitive impairment were both lower in the island group than in the inland group, with statistically significant differences (all *P* < 0.05). See Table 3. After adjustment for age, sex, body mass index, admission NIHSS score, major cerebrovascular risk factors, acute-phase reperfusion therapy, secondary prevention medications after discharge, smoking history, alcohol consumption history, discharge destination, and educational attainment, the association between an island dietary background and a lower risk of poor functional outcome (aOR = 0.58, 95%*CI*: 0.38–0.89, *P* = 0.013), post-stroke depression (aOR = 0.54, 95%*CI*: 0.34–0.86, *P* = 0.009), and post-stroke cognitive impairment (aOR = 0.57, 95%*CI*: 0.37–0.88, *P* = 0.011) remained statistically significant. These associations were consistent in both the sensitivity analysis incorporating source hospital as a covariate (Supplementary Table 3) and the ordinal logistic regression sensitivity analysis using mRS score as an ordinal outcome variable (aOR = 0.63, 95%*CI*: 0.44–0.90, *P* = 0.012; Brant test *P* = 0.318). The corresponding results are presented in Supplementary Tables 3 and 4, respectively.

**Table 3:** Comparison of functional, neurological, and event outcomes at 1 year after stroke among patients with different dietary backgrounds (*n* = 410).

Outcome	Island group (*n* = 210)	Inland group (*n* = 200)	*z*/χ^2^	*P* value
mRS score	2.0 (1.0–3.0)	3.0 (2.0–4.0)	−3.92	< 0.001
Poor functional outcome (mRS > 2), *n* (%)	64 (30.5%)	87 (43.5%)	7.06	0.008
Follow-up NIHSS score	3.0 (1.0–5.0)	4.0 (2.0–6.0)	−3.15	0.002
ΔNIHSS (admission → 1 year)	−3.0 (−5.0–−1.0)	−2.0 (−4.0–0.0)	−2.54	0.011
Stroke recurrence, *n* (%)	14 (6.7%)	18 (9.0%)	0.78	0.377
Post-stroke depression, *n* (%)	40 (19.0%)	62 (31.0%)	7.23	0.007
Post-stroke cognitive impairment, *n* (%)	55 (26.2%)	78 (39.0%)	7.65	0.006

### 3.4 Univariate analysis of major diet-related factors associated with dietary background grouping

The results showed that, compared with low-frequency intake, fish consumption frequencies of 3–4 days/week and 5–7 days/week were both significantly positively associated with the island dietary background (*OR* = 2.58, 95% *CI*: 1.68–3.96, *P* < 0.001; *OR* = 3.12, 95% *CI*: 1.89–5.14, *P* < 0.001). A frequency of shrimp and crab intake ≥1 day/week was also positively associated with the island dietary background (*OR* = 1.82, 95% *CI*: 1.28–2.60, *P* = 0.001). In contrast, red meat intake ≥3 days/week, pickled food intake ≥1 day/week, and fried food intake ≥1 day/week were all negatively associated with the island dietary background (*OR* = 0.52, 95% *CI*: 0.35–0.78, *P* = 0.002; *OR* = 0.60, 95% *CI*: 0.44–0.82, *P* = 0.001; *OR* = 0.66, 95% *CI*: 0.48–0.90, *P* = 0.009). No statistically significant differences were observed for other dietary factors (all *P* > 0.05). These findings suggest that systematic differences in the frequency of intake of different food types exist between island and inland dietary backgrounds and can serve as key features distinguishing the two dietary patterns. See Table 4.

**Table 4:** Univariate logistic regression analysis of major diet-related factors and differences in dietary background groups.

Factor	Category	Island group *n* (%) (*n* = 210)	Inland group *n* (%) (*n* = 200)	OR (95% *CI*)	*P* value
Fish intake frequency	< 1 day/week	20 (9.5%)	48 (24.0%)	1.00	—
	1–2 days/week	52 (24.8%)	68 (34.0%)	1.84 (1.02–3.30)	0.042
	3–4 days/week	86 (41.0%)	58 (29.0%)	2.58 (1.68–3.96)	< 0.001
	5–7 days/week	52 (24.8%)	26 (13.0%)	3.12 (1.89–5.14)	< 0.001
Shrimp and crab intake frequency	< 1 day/week	82 (39.0%)	110 (55.0%)	1.00	—
	≥1 day/week	128 (61.0%)	90 (45.0%)	1.82 (1.28–2.60)	0.001
Shellfish intake frequency	< 1 day/week	94 (44.8%)	102 (51.0%)	1.00	—
	≥1 day/week	116 (55.2%)	98 (49.0%)	1.28 (0.90–1.82)	0.168
Type of cooking oil	Blended oil	70 (33.3%)	90 (45.0%)	1.00	—
	Common vegetable oil†	96 (45.7%)	74 (37.0%)	1.67 (1.08–2.58)	0.021
	ω-3-rich vegetable oil‡	44 (21.0%)	36 (18.0%)	1.57 (0.91–2.70)	0.105
Red meat intake frequency	< 1 day/week	96 (45.7%)	56 (28.0%)	1.00	—
	1–2 days/week	78 (37.1%)	88 (44.0%)	0.61 (0.40–0.94)	0.024
	≥3 days/week	36 (17.1%)	56 (28.0%)	0.52 (0.35–0.78)	0.002
Pickled food intake frequency	< 1 day/week	144 (68.6%)	104 (52.0%)	1.00	—
	≥1 day/week	66 (31.4%)	96 (48.0%)	0.60 (0.44–0.82)	0.001
Fried food intake frequency	< 1 day/week	152 (72.4%)	112 (56.0%)	1.00	—
	≥1 day/week	58 (27.6%)	88 (44.0%)	0.66 (0.48–0.90)	0.009
Soybeans and soy product intake frequency	< 1 day/week	54 (25.7%)	68 (34.0%)	1.00	—
	1–2 days/week	82 (39.0%)	80 (40.0%)	1.29 (0.80–2.08)	0.297

†Common vegetable oils include peanut oil, soybean oil, corn oil, and sunflower oil; ‡ω-3-rich vegetable oils include flaxseed oil.

### 3.5 Multivariable analysis of major diet-related factors associated with dietary background grouping

The results showed that, in the fully adjusted model (Model 2), fish intake frequency remained independently and positively associated with the island dietary background (aOR = 1.36, 95% *CI*: 1.05–1.77, *P* = 0.021), indicating that fish consumption is one of the core features distinguishing island and inland dietary patterns. The use of ω-3-rich vegetable oil as the primary cooking oil source also showed a positive association trend after adjustment, although it did not reach statistical significance (aOR = 1.72, 95% *CI*: 0.92–3.20, *P* = 0.089). The association of red meat intake frequency was attenuated in the multivariable analysis and did not reach statistical significance (*P* > 0.05). No significant associations were observed for other dietary factors after adjustment (all *P* > 0.05). These findings suggest that a dietary pattern characterized by fish and other seafood constitutes a core component of the island dietary background. See Table 5.

**Table 5:** Multivariable logistic regression analysis of the association between major diet-related factors and dietary background groups (*n* = 410).

Variable	Assignment/comparison	Model 1 aOR (95% *CI*)	*P* value	Model 2 aOR (95% *CI*)	*P* value
Fish intake frequency	Per 1-category increase (ordinal)	1.42 (1.13–1.79)	0.003	1.36 (1.05–1.77)	0.021
Other aquatic product intake frequency (shrimp/shellfish)	≥1 day/week vs. < 1 day/week	1.51 (0.98–2.33)	0.062	1.39 (0.88–2.20)	0.156
Type of cooking oil: common vegetable oil†	vs. blended oil	1.52 (0.98–2.36)	0.061	1.44 (0.91–2.28)	0.119
Type of cooking oil: ω-3-rich vegetable oil‡	vs. blended oil	1.90 (1.05–3.42)	0.034	1.72 (0.92–3.20)	0.089
Red meat intake frequency	Per 1-category increase (ordinal)	0.74 (0.60–0.92)	0.006	0.82 (0.64–1.06)	0.132
Pickled food intake frequency	≥1 day/week vs. < 1 day/week	0.70 (0.48–1.02)	0.064	0.77 (0.51–1.16)	0.210
Fried food intake frequency	≥1 day/week vs. < 1 day/week	0.68 (0.46–1.01)	0.056	0.73 (0.48–1.12)	0.152
Soybeans and soy product intake frequency	Per 1-category increase (ordinal)	1.16 (0.95–1.42)	0.149	1.12 (0.90–1.39)	0.308

Model 1: all the above diet-related factors were simultaneously included to identify dietary characteristics independently associated with dietary background grouping. Model 2: further adjusted for age, sex, BMI, admission NIHSS, and other factors that may influence dietary structure reporting or grouping differences based on Model 1. †Common vegetable oils include peanut oil, soybean oil, corn oil, and sunflower oil; ‡ω-3-rich vegetable oils include flaxseed oil.

**Figure 1:**
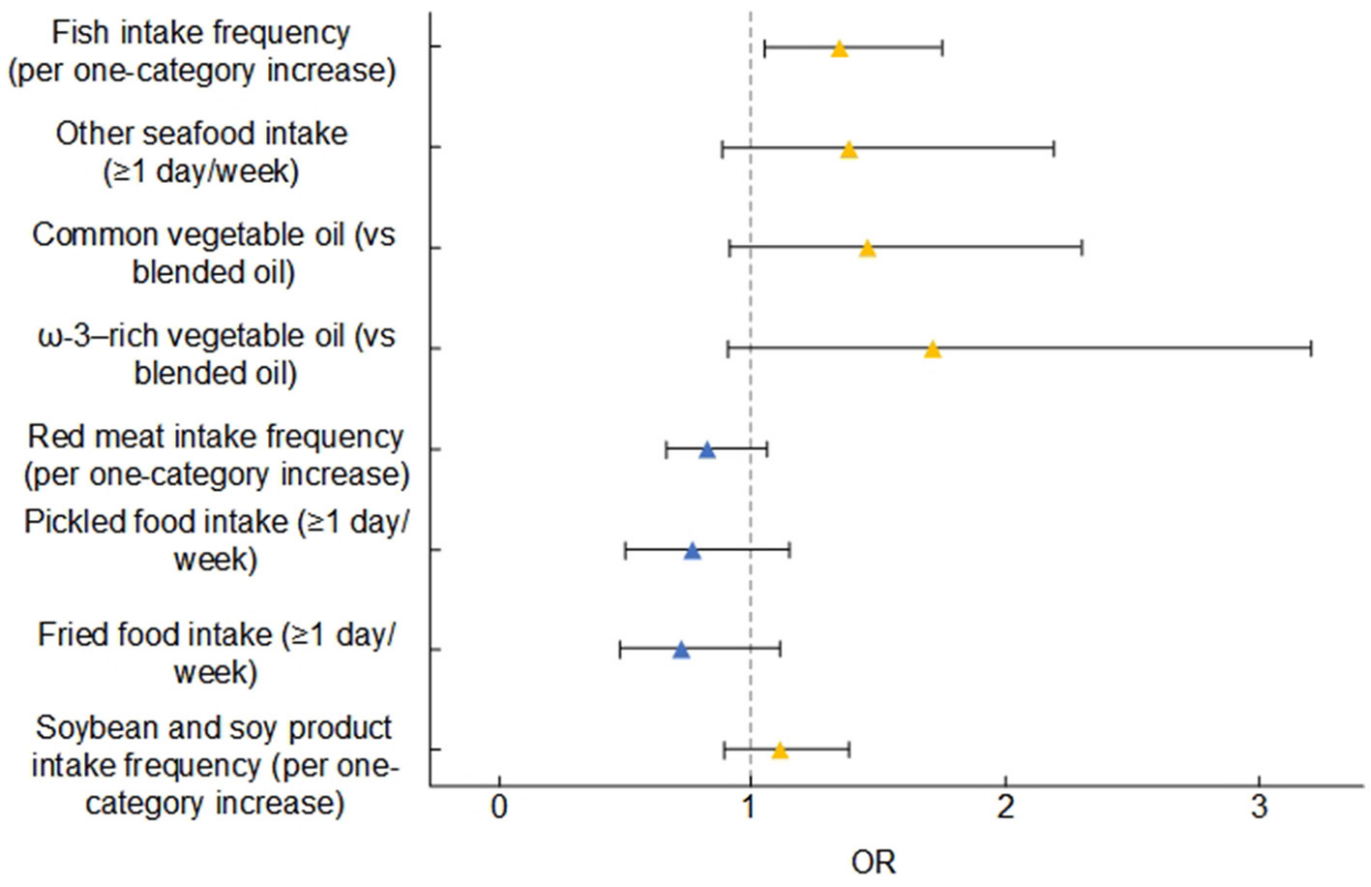
Forest plot of the associations between major diet-related factors and dietary background grouping in the multivariable Logistic regression analysis.

To more intuitively illustrate the direction and stability of the associations of dietary factors in the multivariable model, a forest plot was further used to visualize the results of the multivariable analysis. See Figure 1.

### 3.6 Analysis of ω-6/ω-3 ratio grouping and trend test

After adjustment for age, sex, body mass index, and admission NIHSS score, differences in 1-year clinical outcomes were observed among patients with different ω-6/ω-3 ratio groups, all showing significant trend changes (all *P* for trend < 0.05). As the ω-6/ω-3 ratio increased, the incidence of poor functional outcomes (mRS>2) gradually rose from 20.0% to 53.3%, with an increasing trend in risk (*P* for trend = 0.021). These findings suggest a gradient association between higher ω-6/ω-3 ratios and multiple adverse long-term outcomes. See Table 6. The results of the regression analyses incorporating the ω-6/ω-3 ratio as a continuous variable were consistent with the direction observed in the grouped trend analyses. The corresponding results are presented in Supplementary Table 5.

**Table 6:** Comparison of 1-year clinical outcome risks and trend analysis according to ω-6/ω-3 ratio grouping.

ω-6/ω-3 group	Sample size/*n*	Poor functional outcome (mRS>2) *n* (%)	aOR (95% *CI*)	post-stroke depression *n* (%)	aOR (95% *CI*)	post-stroke cognitive impairment *n* (%)	aOR (95% *CI*)
< 4	30	6 (20.0)	1.00	5 (16.7)	1.00	6 (20.0)	1.00
4–10	40	14 (35.0)	1.68 (0.55–5.10)	12 (30.0)	1.56 (0.52–4.68)	13 (32.5)	1.49 (0.50–4.40)
≥10	30	16 (53.3)	3.05 (0.92–7.98)	14 (46.7)	2.21 (0.73–6.69)	15 (50.0)	2.18 (0.71–6.64)
*P* for trend			0.021		0.038		0.041

The ω-6/ω-3 ratio < 4 group was used as the reference group (reference). The multivariable logistic regression model was adjusted for age, sex, body mass index, and admission NIHSS score. *P* for trend was tested by including the ω-6/ω-3 ratio groups as an ordinal variable in the model.

### 3.7 Correlation analysis between ΔNIHSS and serum polyunsaturated fatty acid profiles

**Figure 2:**
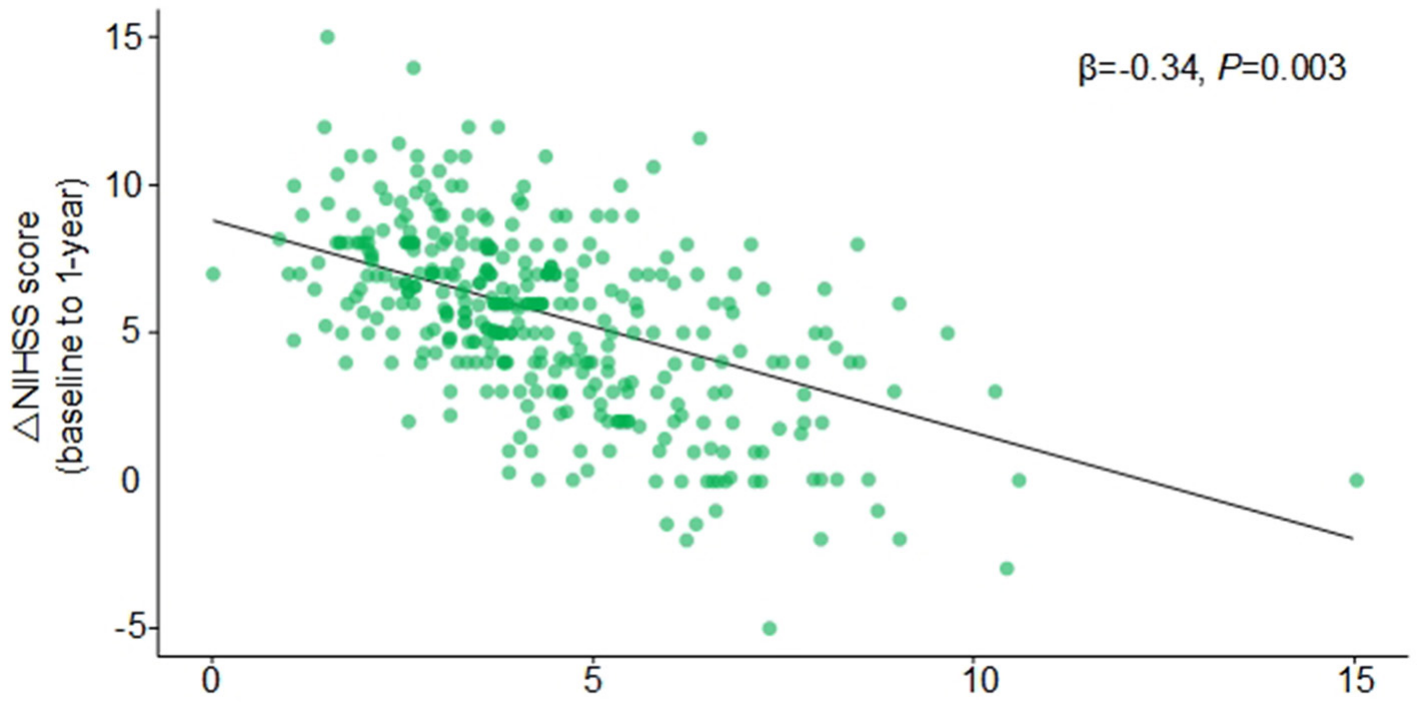
Correlation analysis between serum ω-6/ω-3 ratio and ΔNIHSS score. Negative values indicate improvement in neurological function relative to baseline, and smaller values represent a greater magnitude of improvement.

The results showed that the ω-6/ω-3 ratio was significantly negatively correlated with ΔNIHSS (β = −0.34, *P* = 0.003), indicating that patients with higher ω-6/ω-3 ratios exhibited smaller reductions in NIHSS score at 1-year follow-up, reflecting a lesser degree of neurological improvement. See Figure 2.

## 4 Discussion

Stroke remains one of the leading causes of death and disability in China, with large-artery atherosclerotic cerebral infarction accounting for a relatively high proportion among Asian populations, and with marked heterogeneity in functional recovery as well as emotional and post-stroke cognitive impairment after stroke. Previous studies have suggested that dietary factors are closely related to the atherosclerotic process; however, most have focused on the general population or have not distinguished stroke subtypes, and have mainly relied on questionnaire-based assessments of dietary exposure, making it difficult to directly link diet to *in vivo* metabolic characteristics (18, 19). Based on this, the study compared clinical outcomes among patients with large-artery atherosclerotic cerebral infarction under different dietary backgrounds, and, combined with objective assessment of serum polyunsaturated fatty acid profiles, systematically analyzed the associations of ω-3, ω-6, and their ratio with post-stroke functional outcomes, neurological recovery, as well as emotional and post-stroke cognitive impairment. The results showed that the island characteristic dietary background was associated with more favorable functional recovery and a lower risk of adverse outcomes. Further analyses suggested that differences in serum polyunsaturated fatty acid profiles, particularly the ω-6/ω-3 ratio, may play a role in linking dietary background with clinical outcomes.

In the analysis of dietary structure, multivariable model results indicated that fish intake frequency and the use of plant oil as the primary fat source were positively associated with the island dietary background, whereas red meat intake was negatively associated. Unlike previous studies that mainly focused on individual foods or nutrients, the study further incorporated serum polyunsaturated fatty acid profiles, exploring the continuous pathway from dietary exposure to metabolic phenotype and subsequently to clinical outcomes (20). Related studies have shown that integrating dietary structure with serum polyunsaturated fatty acid profiles may help elucidate the potential mechanisms by which dietary patterns influence stroke prognosis through altering the balance of fatty acids *in vivo* (21). The findings of this study further support the notion that dietary factors may be reflected as quantifiable and longitudinally trackable metabolic characteristics. In secondary prevention and long-term follow-up management after stroke, these results support shifting dietary assessment from general health education to targeted guidance based on fatty acid profiles. For example, in addition to routine pharmacological therapy, patients with higher intake of red meat and highly processed foods or with imbalanced fatty acid profiles may benefit from strengthened dietary structure modification and nutritional follow-up.

Building on the comparison of dietary groups, the study further introduced the serum polyunsaturated fatty acid profile as an objective biomarker and directly quantified it using gas chromatography–mass spectrometry. In the fatty acid detection subgroup, certain associations were observed between fatty acid profile characteristics and multiple clinical outcomes, suggesting that polyunsaturated fatty acid metabolism may be involved in the relationship between dietary background and post-stroke outcomes. The results of regression analyses incorporating the ω-6/ω-3 ratio as a continuous variable were consistent with the direction observed in the grouped trend analyses, further supporting the directionality of these associations. However, because the relevant analyses were conducted in only a subset of patients, the findings should be interpreted as exploratory observations and require further validation in studies with larger sample sizes.

In the outcome analysis, stratification by the ω-6/ω-3 ratio showed that higher ratios were associated with increased risks of poor functional outcomes, post-stroke depression, and cognitive impairment. Previous studies on polyunsaturated fatty acids and cardiovascular and cerebrovascular outcomes have largely focused on the general population, and these associations often attenuate after adjustment for traditional risk factors or overall dietary quality indices, leaving the independent significance of fatty acid ratios in the post-stroke phase unclear (22). In contrast, the study observed a stable association between fatty acid ratios and multidimensional outcomes in a population with relatively homogeneous pathophysiology, namely large-artery atherosclerotic cerebral infarction. In clinical follow-up practice, these findings support the use of the ω-6/ω-3 ratio as an auxiliary indicator to identify patients at higher risk for poor functional recovery, adverse emotional status, or post-stroke cognitive impairment, thereby enabling more targeted planning of follow-up frequency, allocation of rehabilitation resources, and timing of multidisciplinary interventions. In contrast, the present study observed associations between fatty acid ratios and multidimensional outcomes in a population with large-artery atherosclerotic cerebral infarction, a stroke subtype characterized by a relatively homogeneous pathological mechanism. In real-world follow-up practice, these findings suggest that the ω-6/ω-3 ratio may have research value as a potential auxiliary indicator for identifying patients at higher risk of poor functional recovery, adverse emotional status, or post-stroke cognitive impairment. This may facilitate more targeted planning of follow-up frequency, allocation of rehabilitation resources, and timing of multidisciplinary assessment and intervention.

In the continuous analysis of neurological recovery trajectories, an increase in the ω-6/ω-3 ratio showed a stable linear relationship with a smaller magnitude of neurological improvement after stroke. Previous studies have mainly focused on the role of polyunsaturated fatty acids in atherosclerosis development or stroke risk, and clinical evidence regarding their sustained effects during the post-stroke recovery phase remains limited (23). The study demonstrated, through dynamic outcome measures, a potential association between fatty acid ratios and the degree of neurological improvement, suggesting a potential role in the recovery process after stroke. In rehabilitation practice, these findings provide clues for early identification of patients with limited potential for neurological recovery, which may help guide more appropriate decisions regarding rehabilitation intensity, duration, and the early introduction of multidisciplinary interventions.

From a biological perspective, ω-6 and ω-3 polyunsaturated fatty acids share metabolic enzyme systems *in vivo*, and their metabolites exert different effects in the regulation of inflammatory responses, oxidative stress, and vascular endothelial function. Previous studies have shown that ω-6 fatty acid metabolism can generate pro-inflammatory lipid mediators, whereas ω-3 fatty acids contribute to the production of metabolites with anti-inflammatory and pro-resolving properties, with both maintaining a dynamic balance in inflammation regulation. Post-stroke neuroinflammation is considered closely related to secondary injury and functional recovery; therefore, changes in the ω-6/ω-3 ratio may reflect, to some extent, the inflammatory regulatory status of the body and be associated with neural repair processes. In addition, ω-3 fatty acids play important roles in maintaining neuronal membrane fluidity, synaptic plasticity, and neurogenesis, and may also be involved in neurotransmitter regulation and neuroinflammatory processes (24). The study observed that a higher ω-6/ω-3 ratio was associated with increased risks of post-stroke depression and cognitive impairment, which is consistent with previous findings suggesting a link between fatty acid metabolism and neuropsychiatric outcomes, although the underlying mechanisms require further elucidation. Previous studies have suggested that a lower ω-6/ω-3 ratio may help maintain metabolic homeostasis and improve vascular function. In this context, the study observed that patients with an island characteristic dietary background generally exhibited lower ω-6/ω-3 ratios, which is consistent with a dietary pattern characterized by higher intake of aquatic products and lower intake of red meat and processed foods (25). For clinical management, the findings suggest that fatty acid intake patterns and their corresponding metabolic characteristics may be associated with post-stroke outcomes. The serum ω-6/ω-3 ratio may serve as a potential auxiliary indicator for post-stroke risk assessment and prognostic stratification. In the future, it may provide additional information to support post-stroke risk stratification and individualized follow-up management.

However, the study still has room for further development. First, the classification of dietary background was primarily based on retrospective assessment using previous medical records and previously completed dietary survey data. Although the reliability of classification was improved to some extent by including individuals with stable long-term residence and integrating information from multiple sources, exposure misclassification and information bias may still exist. Therefore, caution is warranted when interpreting the relationship between dietary exposure and clinical outcomes. Future studies may incorporate standardized dietary assessment tools or prospective dietary records to achieve more precise quantification of dietary exposure. Second, the study was conducted using data from two centers, which can reflect population characteristics from different regions to a certain extent; however, the geographic coverage remained relatively limited. Future multicenter studies across broader regions, including populations with different dietary structures, are needed to further validate the stability and applicability of the findings in diverse clinical settings. Regarding serum polyunsaturated fatty acid profile analysis, the relevant analyses were conducted in a subgroup of patients with available serum samples and were primarily intended to explore potential associations between different fatty acid profile characteristics and post-stroke outcomes. With further expansion of the sample size and the accumulation of multicenter data, future studies may enable more refined stratified validation of the relationships between specific fatty acid indicators and clinical outcomes, while further improving the stability of effect estimates and the external generalizability of the findings. In addition, the study was mainly based on baseline fatty acid levels. Future studies may incorporate multi-time-point assessments at different stages after stroke and, in conjunction with trajectories of neurological recovery, longitudinally evaluate the dynamic patterns of change in the ω-6/ω-3 ratio and their relationships with clinical outcomes. At the mechanistic level, the study explored potential pathways from the perspective of biological plausibility. Future work may further integrate biomarkers related to inflammatory responses, oxidative stress, and vascular endothelial function to establish a multidimensional analytical framework combining metabolic and inflammatory phenotypes, thereby providing a more systematic understanding of the potential pathways through which fatty acid ratios influence post-stroke neurological recovery and neuropsychiatric outcomes. Finally, because dietary background classification was closely related to long-term residential environment, differences between the island and inland groups may have extended beyond dietary structure to include unmeasured factors such as physical activity, sunlight exposure, social support, and participation in rehabilitation. These factors may influence post-stroke functional, emotional, and post-stroke cognitive impairment; therefore, residual confounding cannot be completely excluded. Future studies should prospectively incorporate relevant lifestyle factors and healthcare resource utilization variables to further validate the independence of the observed associations.

These findings suggest that in large-artery atherosclerotic cerebral infarction, a stroke subtype with marked clinical heterogeneity, reliance solely on traditional risk factors may be insufficient to fully characterize long-term risk. The fatty acid profile, as a potential metabolic phenotype indicator, may provide additional information for post-stroke risk stratification and individualized management. Further studies are needed to validate its stability and clinical utility in larger samples and multicenter populations.

## Data Availability

The raw data supporting the conclusions of this article will be made available by the authors, without undue reservation.
