# Supplementary material for: Dietary background, serum polyunsaturated fatty acid profiles, and 1-year outcomes after large-artery atherosclerotic stroke: a multicenter cohort study
Source: Front Neurol. 2026 Jul 10;17:1864966. doi: 10.3389/fneur.2026.1864966 (PMC13395614; doi:10.3389/fneur.2026.1864966)
Supplement: Supplementary file 1 [file Table_1.docx]

Supplementary Material

**Supplementary Table 1. Comparison of baseline characteristics and outcome distribution between the overall cohort and the fatty acid analysis subgroup**

| Variables | Overall cohort (n=410) | Fatty acid analysis subgroup (n=100) | *t*/*z*/χ² | *P* value |
| --- | --- | --- | --- | --- |
| Age (years) | 72.8 ± 8.2 | 73.1 ± 8.0 | 0.36 | 0.719 |
| Male sex, n (%) | 246 (60.0%) | 61 (61.0%) | 0.03 | 0.864 |
| BMI (kg/m²) | 24.3 ± 3.2 | 24.5 ± 3.1 | 0.52 | 0.603 |
| Education level |  |  | 0.26 | 0.879 |
| Primary school or below, n (%) | 148 (36.1%) | 37 (37.0%) |  |  |
| Middle school, n (%) | 158 (38.5%) | 38 (38.0%) |  |  |
| High school or above, n (%) | 104 (25.4%) | 25 (25.0%) |  |  |
| Admission NIHSS score | 7.0 (5.0–9.0) | 7.0 (5.0–9.5) | −0.41 | 0.681 |
| Acute reperfusion therapy, n (%) | 82 (20.0%) | 20 (20.0%) | 0.01 | 0.981 |
| Internal carotid artery territory, n (%) | 287 (70.0%) | 72 (72.0%) | 0.15 | 0.701 |
| Hypertension, n (%) | 308 (75.1%) | 76 (76.0%) | 0.03 | 0.858 |
| Diabetes mellitus, n (%) | 139 (33.9%) | 35 (35.0%) | 0.05 | 0.827 |
| Coronary artery disease, n (%) | 100 (24.4%) | 23 (23.0%) | 0.08 | 0.773 |
| Atrial fibrillation, n (%) | 61 (14.9%) | 14 (14.0%) | 0.04 | 0.839 |
| Hyperlipidemia, n (%) | 189 (46.1%) | 48 (48.0%) | 0.12 | 0.726 |
| Smoking history, n (%) | 156 (38.0%) | 39 (39.0%) | 0.03 | 0.857 |
| Alcohol use history, n (%) | 102 (24.9%) | 25 (25.0%) | 0.00 | 0.975 |
| Pre-stroke antiplatelet therapy, n (%) | 123 (30.0%) | 30 (30.0%) | 0.00 | 0.981 |
| Pre-stroke statin therapy, n (%) | 119 (29.0%) | 29 (29.0%) | 0.01 | 0.981 |
| Post-discharge antiplatelet therapy, n (%) | 368 (89.8%) | 90 (90.0%) | 0.01 | 0.933 |
| Post-discharge statin therapy, n (%) | 352 (85.9%) | 86 (86.0%) | 0.01 | 0.971 |
| Discharge destination |  |  | 0.18 | 0.914 |
| Home, n (%) | 298 (72.7%) | 73 (73.0%) |  |  |
| Rehabilitation facility, n (%) | 88 (21.5%) | 21 (21.0%) |  |  |
| Nursing home/care facility, n (%) | 24 (5.9%) | 6 (6.0%) |  |  |
| LDL-C (mmol/L) | 2.85 ± 0.78 | 2.82 ± 0.76 | −0.34 | 0.735 |
| HDL-C (mmol/L) | 1.15 ± 0.27 | 1.16 ± 0.28 | 0.29 | 0.772 |
| Triglycerides (mmol/L) | 1.47 (1.05–1.95) | 1.44 (1.02–1.88) | −0.56 | 0.578 |
| Poor functional outcome (e.g., mRS ≥2), n (%) | 168 (41.0%) | 40 (40.0%) | 0.03 | 0.862 |
