# Supplementary material for: Dietary background, serum polyunsaturated fatty acid profiles, and 1-year outcomes after large-artery atherosclerotic stroke: a multicenter cohort study
Source: Front Neurol. 2026 Jul 10;17:1864966. doi: 10.3389/fneur.2026.1864966 (PMC13395614; doi:10.3389/fneur.2026.1864966)
Supplement: Supplementary file 2 [file Table_2.docx]

Supplementary Material

**Supplementary Table 2. Distribution of dietary background groups by recruiting center (n=410)**

| Recruiting Center | Coastal dietary group, n (%) | Inland dietary group n, (%) | Total, n (%) |
| --- | --- | --- | --- |
| Daishan County First People's Hospital | 210 (100.0%) | 0 (0.0%) | 210 (51.2%) |
| Zhejiang Provincial Hospital of Traditional Chinese Medicine | 0 (0.0%) | 200 (100.0%) | 200 (48.8%) |
| Total | 210 (100.0%) | 200 (100.0%) | 410 (100.0%) |

**Note:** All patients in the coastal dietary group were recruited from Daishan County First People's Hospital, and all patients in the inland dietary group were recruited from Zhejiang Provincial Hospital of Traditional Chinese Medicine, resulting in complete correspondence between dietary group assignment and recruiting center. To assess the potential influence of center effects on primary outcomes, the recruiting center was included as a covariate in sensitivity analyses (see Supplementary Table 3).
