# Supplementary material for: Dietary background, serum polyunsaturated fatty acid profiles, and 1-year outcomes after large-artery atherosclerotic stroke: a multicenter cohort study
Source: Front Neurol. 2026 Jul 10;17:1864966. doi: 10.3389/fneur.2026.1864966 (PMC13395614; doi:10.3389/fneur.2026.1864966)
Supplement: Supplementary file 3 [file Table_3.docx]

Supplementary Material

**Supplementary Table 3. Multivariable regression results for primary outcomes under the fully adjusted model and sensitivity analysis with recruiting center as an additional covariate (n=410)**

| Outcome | Main analysis aOR*/β* (95% *CI*) | *P* value | Sensitivity analysis aOR*/β* (95% *CI*) | *P* value |
| --- | --- | --- | --- | --- |
| Poor functional outcome (mRS>2) | 0.58 (0.38–0.89) | 0.013 | 0.63 (0.40–0.98) | 0.041 |
| Post-stroke depression | 0.54 (0.34–0.86) | 0.009 | 0.59 (0.37–0.95) | 0.031 |
| Post-stroke cognitive impairment | 0.57 (0.37–0.88) | 0.011 | 0.62 (0.39–0.99) | 0.045 |
| ΔNIHSS (β) | −0.15 (−0.28–−0.02) | 0.024 | −0.13 (−0.27–−0.01) | 0.038 |

**Note: Main analysis: adjusted for age, sex, BMI, admission NIHSS score, major cerebrovascular risk factors (hypertension, diabetes mellitus, coronary artery disease, atrial fibrillation, and hyperlipidemia), acute reperfusion therapy, post-discharge secondary prevention medications, smoking history, alcohol use history, discharge destination, and education level. Sensitivity analysis: additionally adjusted for recruiting center on top of the main analysis model. The coastal dietary group was used as the reference category for *aOR* estimation. aOR, adjusted odds ratio; *β*, standardized regression coefficient; *CI*, confidence interval; BMI, body mass index; NIHSS, National Institutes of Health Stroke Scale; mRS, modified Rankin Scale.**
