# Supplementary material for: Dietary background, serum polyunsaturated fatty acid profiles, and 1-year outcomes after large-artery atherosclerotic stroke: a multicenter cohort study
Source: Front Neurol. 2026 Jul 10;17:1864966. doi: 10.3389/fneur.2026.1864966 (PMC13395614; doi:10.3389/fneur.2026.1864966)
Supplement: Supplementary file 4 [file Table_4.docx]

Supplementary Material

**Supplementary Table 4. Sensitivity analysis using ordinal logistic regression for mRS score (n=410)**

|  | aOR (95% *CI*) | *P* value |
| --- | --- | --- |
| Main analysis: binary logistic regression (mRS>2) | 0.56 (0.37–0.85) | 0.007 |
| Sensitivity analysis: ordinal logistic regression (mRS 0–6) | 0.63 (0.44–0.90) | 0.012 |
| Proportional odds assumption (Brant test) | — | 0.318 |

**Note: Both models were adjusted for age, sex, BMI, admission NIHSS score, hypertension, diabetes mellitus, coronary artery disease, atrial fibrillation, hyperlipidemia, acute reperfusion therapy, post-discharge secondary prevention medications, smoking history, alcohol use history, discharge destination, and education level. The aOR in the ordinal logistic regression represents the odds of a one-category increase in mRS score. The coastal dietary group was used as the reference category. A Brant test *P* value >0.05 indicates that the proportional odds assumption holds.**
