# Supplementary material for: Dietary background, serum polyunsaturated fatty acid profiles, and 1-year outcomes after large-artery atherosclerotic stroke: a multicenter cohort study
Source: Front Neurol. 2026 Jul 10;17:1864966. doi: 10.3389/fneur.2026.1864966 (PMC13395614; doi:10.3389/fneur.2026.1864966)
Supplement: Supplementary file 5 [file Table_5.docx]

Supplementary Material

**Supplementary Table 5. Association between ω-6/ω-3 ratio as a continuous variable and clinical outcomes (PUFA subgroup, n=100)**

| Outcome | aOR (95% *CI*) | *P* value |
| --- | --- | --- |
| Poor functional outcome (mRS>2) | 1.68 (1.12–2.52) | 0.012 |
| Post-stroke depression | 1.54 (1.03–2.31) | 0.036 |
| Post-stroke cognitive impairment | 1.52 (1.01–2.28) | 0.044 |

**Note: aOR represents the odds ratio per 1-SD increase in ω-6/ω-3 ratio. Multivariable logistic regression models were adjusted for age, sex, BMI, admission NIHSS score, hypertension, diabetes mellitus, coronary artery disease, atrial fibrillation, hyperlipidemia, acute reperfusion therapy, post-discharge secondary prevention medications, smoking history, alcohol use history, discharge destination, and education level. BMI, body mass index; NIHSS, National Institutes of Health Stroke Scale; mRS, modified Rankin Scale; aOR, adjusted odds ratio; *CI*, confidence interval; SD, standard deviation.**
